# Is There a “Blind Spot” in Point-of-Care Testing for Residual Heparin After Cardiopulmonary Bypass? A Prospective, Observational Cohort Study

**DOI:** 10.1177/1076029620946843

**Published:** 2020-08-14

**Authors:** Saskia Wand, Daniel Heise, Nadine Hillmann, Christian Bireta, Anselm Bräuer, Nicolas von Ahsen, Michael Quintel

**Affiliations:** 1 Department of Anesthesiology, University Medical Center Goettingen, Goettingen, Germany; 2 Department of Anesthesiology, Ruhr-University Bochum, St. Josef- and St. Elisabeth Hospital, Bochum, Germany; 3 Department of Thoracic, Cardiac and Vascular Surgery, University Medical Center Goettingen, Germany; 4 Department of Clinical Chemistry, University Medical Center Goettingen, University Medical Center Goettingen, Goettingen, Germany

**Keywords:** autologous pump blood, residual heparinization, thromboelastometry, heparinase, heparin/protamine titration

## Abstract

Identifying the cause of a bleeding complication after cardiac surgery can be crucial. This study sought to clarify whether the application of unprocessed autologous pump blood influences anti-factor Xa activity after cardiac surgery and evaluated 2 point-of-care methods regarding their ability to identify an elevated anti-factor Xa activity at different timepoints after cardiopulmonary bypass. Anti-factor Xa activity, heparin/protamine titration and the clotting time ratio of thromboelastometry in the INTEM and HEPTEM were measured at baseline (T1), after the application of protamine (T2) and after the complete application of autologous pump blood (T3). Anti-factor Xa activity decreased significantly between T2 and T3 as well did the absolute number of patients with an elevated anti-factor Xa activity. Receiver Operating Curve analyses were performed for both point-of-care methods. At T2 neither could identify patients with an elevated anti-factor Xa activity, while both methods were able to do so at T3 with high sensitivity and specificity. This difference suggests that an interference in the detection of residual heparinization with point-of-care methods exists right after the application of protamine, which seems to subside after a short time span. Nevertheless, results of point-of-care testing for residual heparinization after cardiopulmonary bypass need to be interpreted considering the protamine-heparin ratio and the timepoint of protamine administration.

## Introduction

Postoperative bleeding is a devastating complication for patients undergoing cardiac surgery with cardiopulmonary bypass (CPB). A significant number of patients are affected by this complication despite improvements in the management of bleeding over the past several years. Postoperative bleeding and the subsequent transfusion of packed red blood cells have adverse effects such as higher rates of wound and systemic infections, longer ICU and postoperative hospital stays and overall increased mortality.^1–3^

There are numerous causes of postoperative bleeding, including hypothermia, impaired platelet function and hyperfibrinolysis.^4^ The residual anticoagulatory effect of heparin may also be a reason of excessive postoperative blood loss, possibly caused by insufficient antagonization using protamine, heparin-rebound or the application of heparin-containing unprocessed autologous pump blood at the end of the operating procedure.

Point-of-care methods (POC) have been integrated in the management of perioperative hemostasis in cardiac surgery, since algorithm-based hemotherapy guided by POC methods leads to lower transfusion rates of allogenic blood products.^5,6^ However, there is evidence, that rotational thromboelastometry might not always generate conclusive results concerning a potential heparin effect after cardiac surgery.^7^

This study sought to clarify whether the application of unprocessed autologous pump blood influences anti-factor Xa activity after cardiac surgery. Additionally, it was the aim to evaluate different available POC methods with regard to their sensitivity and specificity for the identification of an elevated anti-factor Xa activity at different timepoints after CPB.

## Methods

### Study Population

The study was registered at the German Clinical Trials Register (DRKS) on Dec 5, 2012 with the number DRKS00003593 and approved by the local ethics committee. After approval 100 consecutive adult patients undergoing elective cardiac surgery with CPB were enrolled into this prospective, observational trial. Patients were screened for eligibility 1 day prior to surgery, and written informed consent was obtained. Exclusion criteria were emergency procedures and contraindications for the use of protamine or heparin, e.g. a known heparin-induced thrombocytopenia or known allergies. Patients who received low-molecular-weight heparin (LMWH) in prophylactic or therapeutic dosages preoperatively were also excluded because of possible interferences with the anti-factor Xa activity test.

A loading dose of unfractionated heparin (300 IU/kg) was administered before cannulation for CPB and activated clotting time (ACT) was measured using a Hemochron^®^ Jr. Signature (Keller Medical, Bad Soden, Germany). An additional bolus of 5,000 IU of unfractionated heparin was administered if ACT was less than 400 seconds. The priming volume (1500 ml) of the heart-lung machine (Maquet Jostra HL20, SOMA Technology, Inc., Bloomfield, IN, USA) also included 7.500 IU of unfractionated heparin. Core body temperature was maintained between 32°C and 36°C during CPB. Unfractionated heparin was antagonized by protamine chloride based on ACT measurements after CBP termination. All patients received 1 g of tranexamic acid as prophylaxis against hyperfibrinolysis with the CPB priming volume and 1 g of tranexamic acid after CPB. Autologous pump blood was administered postoperatively according to the patients’ need for volume therapy in the ICU.

### Blood Sampling and Standard Laboratory Coagulation Parameters

All blood samples were drawn through an arterial line at 3 different time points: (T1) Prior to the operating procedure after induction of general anesthesia, (T2) 15 minutes after the administration of protamine after the termination of CPB, and (T3) directly after the application of autologous pump blood was completed in the ICU. A volume of 5 ml of blood was drawn and discarded before sampling.

Standard laboratory parameters for coagulation screening were obtained at all 3 time points, including platelet count, prothrombin time (PT), activated partial thromboplastin time (aPTT) and fibrinogen concentration.

### Anti-Factor Xa Activity

Citrated blood was centrifuged at 3000 rpm for 10 minutes immediately after sampling, and the resulting plasma was pipetted into a test tube and centrifuged again for 10 minutes at 3000 rpm to eliminate residual platelets. The supernatant was transferred into a test tube and frozen at -20°C until the assays were performed in batches.

Measurements were performed by the Department for Clinical Chemistry using the Berichrom^®^ Heparin assay (Dade Behring, Marburg, Germany). The results were noted in IU/mL.

### Point of Care Methods

#### Heparin/protamine titration (HPT)

The Hepcon^®^ Heparin Management System (HMS) Plus (Medtronic Inc., Minneapolis, MN, USA) required 400 µl blood for each test. The device followed an automated protocol for the HPT (Heparin Assay Red). HPT was performed according to the manufacturer’s instructions using HPT cartridges containing 0 to 0.9 mg/kg protamine.

#### Rotational thromboelastometry

The ROTEM^®^ analyzer (TEM International, Munich, Germany) was used for thromboelastometry. Tests were performed using cups and pins provided by TEM International. Quality control was ensured by regular measurements with ROTROL^®^ quality control serum (TEM International).

We conducted an intrinsically activated test to detect heparin effects with the addition of heparinase (HEPTEM) and without (INTEM) according to the manufacturer’s recommendations. (INTEM: 20 µl CaCl_2_ 0.2 mol/l, 20 µl thromboplastin-phospholipid and 300 µl blood; HEPTEM: additional 10 µl of heparinase).

The clotting times (CT) of both tests were recorded in seconds, and the results were compared for the detection of heparin effects. In the presence of heparin, the CT in the INTEM test will be longer than the CT in the HEPTEM test with added heparinase to neutralize heparin effects in the test. Since the manufacturer gives a normal variance in CT measurements of 10%, the INTEM: HEPTEM CT ratio was considered normal in between 0.9 and 1.1. Values >1.1 were interpreted as possible effects of heparin, values <0.9 as possible interference of protamine with the CT HEPTEM.

### Statistical Analysis

Due to the exploratory nature of the study no sample size calculation was performed. A sample size of 100 patients was considered sufficient by the local ethics board to demonstrate a clinically meaningful difference.

Descriptive statistics were used to summarize study demographics. Normal distribution was examined using the Kolmogorov-Smirnoff test. Normally distributed data are presented as mean with standard deviation (SD).

A Fisher exact test was used to compare proportions. Groups were compared using the Wilcoxon, Mann-Whitney or t-test as appropriate.

Receiver operating characteristic (ROC) curves were calculated for both POC methods to predict residual heparin concentrations >0.3 IU/mL. A p value of less than 0.05 was considered statistically significant.

ROC curves were generated using MedCalc^®^ (MedCalc Software, Mariakerke, Belgium). All remaining statistical analyses were performed using Statistica 13.3^®^ (Tibco Software Inc., Palo Alto, CA, USA).

## Results

Of the 100 patients enrolled to the study, 3 were excluded retrospectively due to an increased anti-factor Xa activity at baseline (T1) and a suspected preoperative LMWH exposition. The sociodemographic and clinical characteristics of the remaining 97 patients are displayed in Table 1, the course of standard laboratory parameters is shown in Table 2.

**Table 1.** Sociodemographic and Clinical Characteristics.

Age [years]	66.8 (9.9)
BMI [kg/m2]	28.0 (4.7)
Gender [male/female; n]	75/22
Procedure [n (%)]	Coronary artery 63 (64.9) Valve surgery 14 (14.5) Combination 20 (20.6)
Mean heparin/kg [IE/kg]	306 (18.97)
Mean protamine/kg [IE/kg]	288 (40.15)
Autologous pump blood [ml]	1120 (293)

Values presented as mean (SD).

**Table 2.** Standard Laboratory Parameters.

	T1	T2	T3
PT (%)	101.4 (13.1)	62.3 (14.6)*	69.9 (12.4)^#^
apTT (sec)	29.7 (18.0)	41.0 (20.2)*	44.1 (22.8)
Fibrinogen (mg/dl)	404.5 (135.2)	192.3 (70.4)*	220.4 (77.3)^#^
Platelet count (/nl)	250 (87.2)	155 (56.3)*	161 (60.8)^#^

Values given as mean (SD); *indicating significant difference between T1 and T2; ^#^indicating significant difference between T2 and T3; PT, prothrombin time; apTT, activated partial thromboplastin time.

Before the application of the autologous pump blood (T2) 11 (11.7%) patients showed an anti-factor Xa activity >0.3 IU/ml. This number decreased to n = 5 (5.1%) after the application of 1120 (± 293) ml of autologous pump blood at T3. Mean anti-factor Xa activity decreased significantly from 0.11 IU/ml at T2 to 0.06 IU/ml at T3 (p = 0.009). The mean interval between T2 and T3 was 92 minutes (± 8.8).

### Detection of Residual Heparinization with POC-Methods

**Figure 1.**
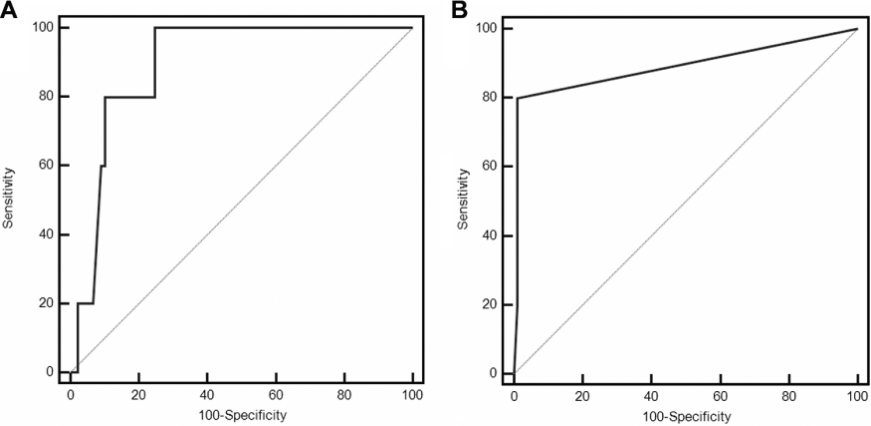
Receiver operating curve illustrating the ability to identify an anti-factor Xa activity >0.3 IU/ml for (A) INTEM: HEPTEM CT ratio with an AUC 0.894 (p<0.0001) and (B) heparin-protamine titration with an AUC 0.891 (p = 0.0001).

ROC analyses for both POC methods failed to yield an AUC significantly different from 0.5 at 15 minutes after the administration of protamine (T2). After the application of autologous pump blood was completed (T3) ROC analyses showed an AUC significantly different from 0.5 for their ability to detect an anti-factor Xa activity >0.3 IU/ml (Figure 1) for both methods with an AUC of 0.894 (p < 0.0001) for the INTEM: HEPTEM CT ratio and an AUC of 0.891 (p = 0.0001) for the HPT, respectively. Cut-off points for the best Youden index with corresponding sensitivity and specificity are given in Table 3.

**Table 3.** Sensitivity and Specificity for ROC Analyses of POC Methods.

	Cut-off with best Youden Index	p-value	Sensitivity [%] (95% CI)	Specificity [%] (95% CI)
INTEM: HEPTEM CT Ratio	>1.18	<0.0001	100.0 (47.8-100.0)	75.28 (65.0-83.3)
Heparin-Protamine Titration	>0 IE Protamine	0.0001	80.0 (28.4-99.5)	98.89 (94.0-100.0)

Values given with 95% confidence interval (95% CI).

### Protamine-Heparin Ratio and INTEM: HEPTEM CT Ratio

**Figure 2.**
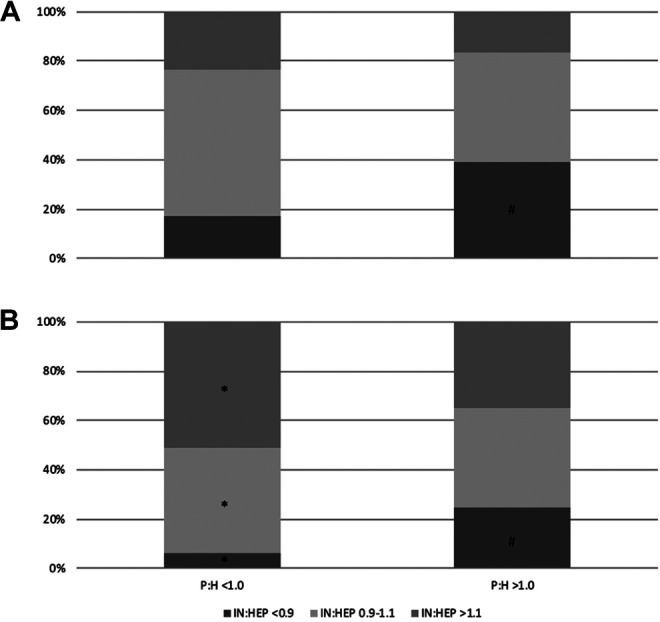
Incidence of different INTEM: HEPTEM CT ratios with respect to Protamine: Heparin (P: H) ratio (A) at T2 and (B) at T3; *indicating a significant difference when compared to T2; ^#^indicating a significant difference compared to P: H ratio <1.0.

The Protamine: Heparin (P: H) ratio had a significant influence on the results of the INTEM: HEPTEM CT ratio. Patients with a Protamine: Heparin (P: H) ratio >1.0 displayed an INTEM: HEPTEM CT ratio <0.9 significantly more often, both at T2 and T3 (17.1% vs. 38.8% (T2), p = 0.04 and 6.6% vs. 25.0% (T3), p = 0.03) (Figure 2). Additionally, the incidence of an INTEM: HEPTEM CT ratio <0.9 significantly decreased between T2 and T3 for patients with a P: H ratio <1.0 (17.1% vs. 6.6%, p = 0.038)(Figure 2), while the number of patients with an INTEM: HEPTEM CT ratio >1.1 significantly increased (23.7% vs. 51.3%, p = 0.0004) (Figure 2).

Though they trended in a similar direction, the changes in the distribution of INTEM: HEPTEM ratio were not significant between T2 and T3 in patients with a P: H ratio >1.0 (Figure 2).

## Discussion

This study evaluated possible effects due to the application of autologous pump blood on the anti-factor Xa activity and the ability of different POC methods to detect a residual heparinization after cardiac surgery.

The application of unprocessed autologous pump blood did not influence the anti-factor Xa activity in patients after cardiac surgery. In fact, we observed a significant decrease over time in anti-factor Xa activity despite the autologous transfusion. This supports evidence found by Iyer et al., that the application of unprocessed pump blood does not lead to an impairment of coagulation due to the infusion of residual heparin.^8^

Though residual heparinization was not commonly found after cardiac surgery, we did see patients in the present study with clearly elevated anti-factor Xa activity after CPB indicating residual heparinization. However, it was not the aim of the study to link an elevated anti-factor Xa activity to possibly increased postoperative blood loss or hemorrhagic complications, since the number of patients would have been far too small to evaluate such relationships. Our interest was solely the evaluation of different POC methods with regard to their ability to identify an elevated anti-factor Xa activity in the postoperative phase after CPB.

The negative effects of excess protamine on the coagulation system have been demonstrated repeatedly.^9–13^ Therefore, it is important to ensure for a high specificity in a method used for the diagnosis of residual heparinization to minimize the number of false positive tests, since these patients are at risk to receive protamine unnecessarily with all the negative consequences mentioned above.

The results of the present study indicate that 2 POC methods currently used for the identification of residual heparinization might be subject to interference in the period directly after the administration of protamine for the termination of CPB. This interference seems to decrease significantly over time, indicating that the INTEM: HEPTEM CT ratio and the HPT can be used reliably for the evaluation of a possible residual heparinization in the postoperative period after CPB, but not immediately after the reversal of heparin for the termination of CPB.

Concerning the HPT, which has not been integrated as a standard method in POC-based algorithms for the management of bleeding complications in cardiac surgery, this might be of less consequence. It has mainly been studied with respect to its benefits in the protamine dose determination for the reversal of heparinization after CPB.^14–16^ In the postoperative period after CPB opinions differ regarding the value of the HPT.^5,17,18^ One reason for these different opinions could possibly lie in the influence of an interference in the period after the termination of CPB. Although the HPT is not widely used, this fact needs to be evaluated further for a better understanding of the limitations of this method and to define periods after CPB, where its use might not be recommendable.

In contrast, the INTEM: HEPTEM CT ratio is an integrated part of POC-based management algorithms for bleeding complications after cardiac surgery.^5^ These algorithms give clear guidance and recommendations of action, when the INTEM: HEPTEM CT ratio is elevated. Thus, a possible interference with this method could lead to more therapeutic consequences for patients after CPB. Our results support evidence found by Meesters et al., that thromboelastometry with the addition of heparinase is subjected to interference directly after the reversal of heparinization with protamine at the end of CPB.^7^ The most likely explanation for this phenomenon is a possible liberation of protamine due to degradation of heparin by heparinase resulting in an elongation of the CT by protamine.^11,12,19^ Additionally, results of the present study suggest that excess protamine due to P: H ratios >1.0 could have a negative influence on INTEM: HEPTEM CT ratio measurements as well. This is in line with measurements in blood samples from healthy volunteers, that showed an influence of increasing P: H ratios on the HEPTEM CT.^7^

As a clinical implication from the results of the present study, we believe it is necessary to complement existing POC algorithms with regard to the interpretation of INTEM: HEPTEM CT ratio, taking the timepoint of protamine administration and the P: H ratio into consideration.

These modifications cannot be drawn from the existing data and further studies are needed. Especially, since more detailed information on the decrease of interference over time would be necessary for these modifications. One possibility could be the combined use of different POC methods to increase sensitivity and specificity for the detection of residual heparinization. A method for this purpose could be the activated clotting time (ACT), which is widely used for the management of heparinization during CPB. Its limitations in the postoperative period have been shown.^20,21^ Nevertheless, it is a method readily available and it could be a useful complementation of the INTEM: HEPTEM CT ratio, since a normal ACT might confirm the validity of a normal INTEM: HEPTEM CT ratio in the period directly after the termination of CPB.

### Limitations

The present study has some limitations. First, the interval between T2 and T3 was variable in length as it was defined by the duration of application of the autologous pump blood. The need for volume therapy was determined solely by the attending physician and the clinical needs of the individual patient. Therefore, no conclusions can be drawn from the existing data, regarding the minimum interval needed for the interference in INTEM: HEPTEM CT ratio to subside. Especially for the complementation of POC algorithms, this is an essential information, which needs to be explored in further studies with higher patient numbers than were evaluated in the exploratory design of the present study. Second, we did not perform baseline measurements for INTEM: HEPTEM CT ratio. A potential exposition to heparin preoperatively was evaluated by a baseline anti-factor Xa activity. With the knowledge that some patients already display an elongated HEPTEM CT before exposition to protamine and the fact that there seems to be a rather high variation in results within the same patient for CT measurements, this could have been an interesting aspect for a better understanding of our data.^7^ Third, we did not collect any data concerning perioperative blood loss in our patients. We are aware, that abnormal findings in laboratory measurements alone are of no consequence in a patient that displays no signs of an increased bleeding tendency, but the study was designed to provide more information on the sensitivity and specificity of the INTEM: HEPTEM CT ratio and the HPT for the identification of an elevated anti-factor Xa activity in the phase after the termination of CPB.

## Conclusion

In conclusion, the administration of autologous pump blood does not lead to an increase in anti-factor Xa activity after CPB. Regardless of the cause, some patients display an elevated anti-factor Xa activity after the termination of CPB. There seems to be a “blind spot” in the detection of this impairment of coagulation with POC methods right after the application of protamine. The importance and superiority of POC methods in algorithm-based hemotherapy after cardiac surgery have been shown.^5,6^ Though the interference due to protamine seems to subside after a rather short time span, we believe that these algorithms might need modifications to ensure their ability to reliably identify residual heparinization in the period right after the termination of CPB.
